# Anisotropic Effects in Local Anodic Oxidation Nanolithography on Silicon Surfaces: Insights from ReaxFF Molecular Dynamics

**DOI:** 10.1021/acs.langmuir.4c01129

**Published:** 2024-07-15

**Authors:** Jian Gao, Wenkun Xie, Xichun Luo, Yi Qin, Zhiyong Zhao

**Affiliations:** Centre for Precision Manufacturing, Department of Design, Manufacturing and Engineering Management, University of Strathclyde, Glasgow G1 1XJ, U.K.

## Abstract

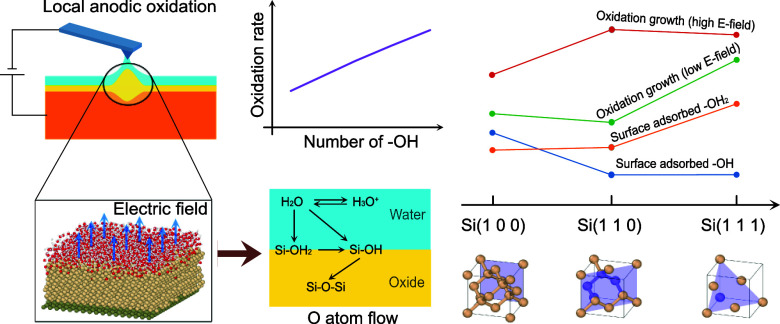

Fully understanding the anisotropic effect of silicon surface orientations in local anodic oxidation (LAO) nanolithography processes is critical to the precise control of oxide quality and rate. This study used ReaxFF MD simulations to reveal the surface anisotropic effects in the LAO through the analysis of adsorbed species, atomic charge, and oxide growth. Our results show that the LAO behaves differently on silicon (100), (110), and (111) surfaces. Specifically, the application of an electric field significantly increases the quantity of surface-adsorbed −OH_2_ while reducing −OH on the (111) surface, and results in a higher charge on a greater number of Si atoms on the (100) surface. Moreover, the quantity of surface-adsorbed −OH plays a pivotal role in influencing the oxidation rate, as it directly correlates with an increased formation rate of Si–O–Si bonds. During bias-induced oxidation, the (111) surface appears with a high initial oxidation rate among three surfaces, while the (110) surface underwent increased oxidation at higher electric field strengths. This conclusion is based on the analysis of the evolution of Si–O–Si bond number, surface elevation, and oxide thickness. Our findings align well with prior theoretical and experimental studies, providing deeper insights and clear guidance for the fabrication of high-performance nanoinsulator gates using LAO nanolithography.

## Introduction

Local anodic oxidation (LAO) nanolithography is a promising nanofabrication technique to accommodate fast and flexible prototyping of functional nanostructures.^1−5^ It relies on the controlled oxidation of conductive surfaces in anodic solutions, which is induced by an enhanced local electric field created by applying a bias between the nanoscale probes/electrodes and substrate.^6^ Compared with other nanomanufacturing techniques, such as optical lithography, electron beam lithography, and nanoimprint lithography, LAO has advantages in atomic-level resolution, direct surface patterning ability, and low instrument cost.^7,8^ It has found broad applications in the manufacturing of quantum devices,^9^ synaptic devices,^10^ metasurfaces,^11^ memristors,^12^ transistors,^13^ Schottky junctions,^14^ gas sensors,^15^ photoluminescence enhancement,^16^ and optical devices.^17^

Despite the promise of this approach, several challenges impede its further development and industrial implementations. First, oxides produced by LAO are of low quality due to their lower density and dielectric strength, compared to oxides formed by dry oxidation. Specifically, LAO-derived oxide exhibits a density of 2.05 g/cm^3^, lower than the 2.27 g/cm^3^ density of thermally oxidized silicon.^18,19^ These oxides also contain a considerable amount of water, approximately 4–5% by mass.^20,21^ As a result, LAO-generated oxide layers can detrimentally affect the function and performance of silicon-based electronics, particularly under high temperatures, limiting their direct use in transistors. To implement these oxides, additional postannealing and surface modification steps are necessarily required to enhance their compatibility and functionality.^22^ Moreover, the anisotropic effects, pivotal in nanoelectronic device fabrication,^23^ received limited attention in LAO processes. The distinct properties of various silicon surface orientations significantly impact their functionality. For instance, silicon (100) orientation is favored for complementary metal-oxide-semiconductor (CMOS) manufacturing due to its advantageous interfacial qualities,^24^ while silicon (111) surface is selected for bipolar transistors owing to its densely packed plane,^25−28^ and silicon (110) surface is preferred for fabricating low-dimensional structures such as nanowires.^29,30^ Thus, understanding the oxide formation on these different surface orientations is vital for refining LAO processing parameters,^26^ further facilitating the development of these silicon-based electronic devices using LAO nanolithography.

Reactive force field molecular dynamics (ReaxFF MD) simulation has emerged as a preferred approach for investigating the intricate reaction mechanisms in various nanomanufacturing processes at nanometric and even atomic scales.^31,32^ It is capable of elucidating bonding interactions, chemical compositions, and atomic dynamics while offering reduced computational cost compared to first-principles methods.^33−35^ ReaxFF MD simulations have been employed to extensively study silicon surface wet oxidation processes, which are modeled in a similar scenario to that of the LAO process. Pamungkas et al.^36^ examined the initial stage of silicon (001) oxidation by water, revealing that hydrogen atoms in water bond with silicon more readily than oxygen atoms in O_2_. They also found that the repulsion between water molecules and their fragments facilitated the dissociation of both water and hydroxyl decomposition on silicon surfaces. Wen et al.^37^ investigated interactions between water and silicon at various temperatures and surface orientations. They identified two forms of water adsorption mechanisms—molecular and dissociative adsorption—with their dominance varying depending on the surface. Additionally, they found that higher temperatures significantly enhance oxidation. Yuan et al.^38−41^ conducted extensive ReaxFF MD simulation studies on the oxidation behaviors of silicon surfaces with various chemical grafts. Their analysis revealed that silicon surfaces oxidize via peroxy-like (H_2_O_2_) structures, with different chemical grafts having distinct effects on the oxidation process. The insights gained from these studies are expected to aid in the optimization of semiconductor device manufacturing. In contrast, ReaxFF MD simulation studies on LAO have only been reported in recent years. Hasan et al.^42^ investigated bias-induced oxidation mechanisms, observing similar water adsorption mechanisms as Wen et al.^37^ and demonstrating the acceleration effects of increased electric field strength and humidity levels. Hasan et al.^43^ also studied the effects of surface orientation during the bias-induced oxidation process, finding that oxide growth varied across different surfaces and was influenced by the electric field strength. However, both of the existing LAO simulation reports overlooked the presence of the surface passivation layer on silicon, a critical factor influencing oxide growth and device functionality.^22,44−46^ Additionally, their analysis methods typically involve straightforward evaluations without distinguishing between the types of bonds/species within the reaction system or performing comprehensive analyses of oxide growth. As a result, detailed explanations of oxidation behaviors and their anisotropic effects on silicon surface orientations in the LAO process are lacking. This represents a key knowledge gap for quality control of oxide films in the LAO process.

In this context, we performed the ReaxFF MD simulation to study the microscopic process of bias-induced oxidation on silicon surfaces with different orientations at (100), (110), and (111). By performing detailed analysis of surface-adsorbed species, chemical bonds, charge states, and oxide growth rate, more insights into LAO mechanisms and anisotropic effects were presented.

## Methods

**Figure 1:**
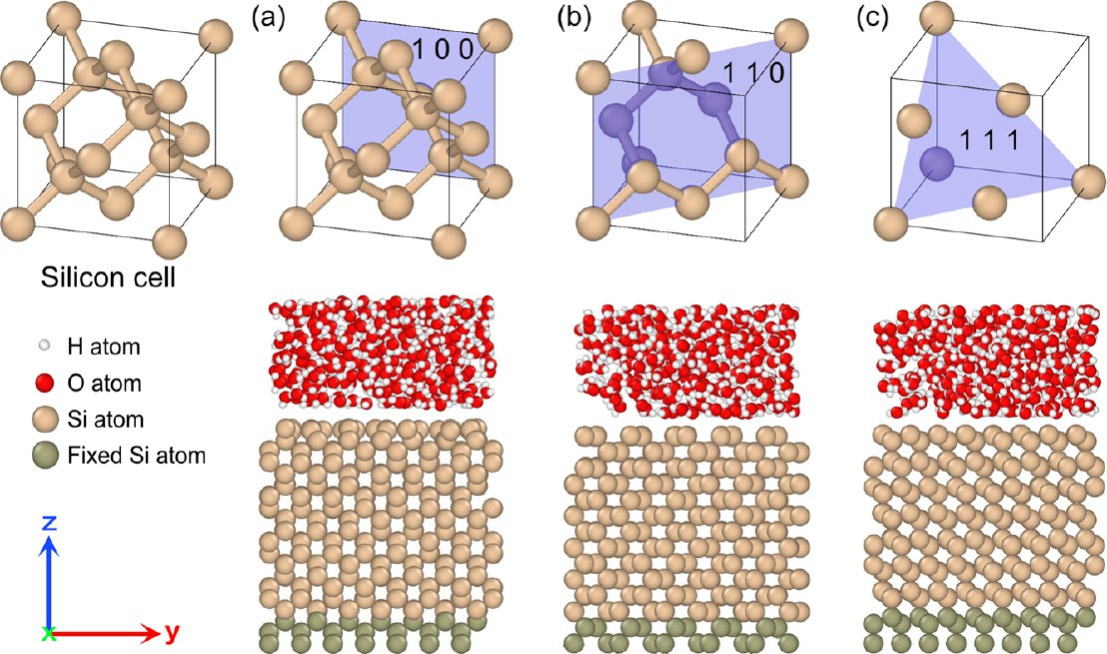
Schematics of simulation models for water layers and silicon substrates with surface orientations at (100), (110), and (111).

In this work, ReaxFF MD simulation models of silicon/water with an applied electric field were established following the procedure in previous reports.^47,48^Figure 1 shows the simulation models for surface passivation simulation with parameters listed in Table 1. Crystalline silicon substrate models were obtained using a large-scale atomic/molecular massively parallel simulator (LAMMPS)^49^ with a lattice constant of 5.31 Å. Randomly distributed water models were created using PACKMOL,^50^ which has a density of 1 g/cm^3^. Water models were set with the same lateral dimension as the silicon substrate and the same thickness of 1.3 nm. This value was selected by referring to a previous study conducted under similar simulation conditions,^47^ which demonstrated that this thickness is sufficient to facilitate noticeable bias-induced oxidation. Before assembling silicon and water models, the silicon substrate models were relaxed at room temperature using canonical ensemble (*NVT*) with Berendsen thermostat for 1 ns. After preparing these models, we conducted a surface passivation simulation for 1 ns. Both our simulation and previous studies^47^ have shown that a 1 ns simulation period allows the reaction system to reach a relatively static state, where major species exhibit no significant changes. A similar strategy has also been used in another MD simulation study involving preoxidation layers.^45^ Then, to simulate the bias-induced oxidation on different silicon surfaces, the initial models were prepared based on the passivation results containing passivated oxide layers on the silicon surfaces.

**Table 1:** Parameters for Simulation Models of Silicon (100), (110), and (111) Substrates

surface orientations		(100)	(110)	(111)
substrate size (nm)		2.628 × 2.628 × 2.522	2.655 × 2.628 × 2.441	2.601 × 2.628 × 2.453
number of Si atoms		980	980	952
number of H_2_O molecules		276	279	273
surface atom density (/nm^2^)		7.093	10.031	8.190
interlayer distances (nm)	first:	0.133	0.188	0.077
	second:	0.133	0.188	0.230
	third:	0.133	0.188	0.077

Periodic boundary conditions were introduced in the *x* and *y* directions to reduce the boundary effects. Along the *z*-axis, a fixed condition was applied with a reflective wall placed on the top of the water layer to prevent undesired oxidation on the bottom silicon layer. The bottom three layers of Si atoms were fixed to prevent the overall movement. The simulation of surface passivation and bias-induced oxidation was performed in the *NVT* ensemble, depending on the number of particles, volume, and the absolute temperature at 300 K with the damping constant of 10 fs. A Verlet algorithm was adopted to integrate the atom trajectories.

In this work, the interactions between atoms are described by the ReaxFF force field developed by Wen et al.^51^ This force field combines the Si/Ge/H force field^52^ and the water force field^53^ and has been extensively validated in previous studies of the water–silicon interactions,^51^ chemical mechanical polishing of silicon,^54^ and tribochemical wear at the Si/SiO_2_ interface in an aqueous environment.^37^ In addition, in the research by Hasan,^55^ this force field was validated by comparing the cohesive energy and surface energy obtained from the ReaxFF MD simulation with those obtained from other potentials, density functional theory, and experiments. The results showed good agreement, further confirming the accuracy and reliability of the force field. To facilitate ReaxFF MD simulation, the charge equilibration (QEq) model was used to equilibrate the charge of simulation models at each time step.^56,57^ To consider the influence of external electrical field in the simulations, this study employed a modified LAMMPS code.^49,58^ The modification was based on the formula proposed by Chen and Martinez,^59^ which takes into account atom polarization, charge conservation, and electronegativity differences using the charge-fluctuation model. In order to calculate electrostatic properties, such as multipolar moments and polarizability, the electric field is integrated into the Coulomb energy within the ReaxFF potential. A detailed description of this implementation can be found in the paper of Assowe et al.^58^

Finally, detailed and comprehensive analysis and postprocessing were performed for simulation results through different aspects, including particle/bond type and number, atomic charge, and their distributions. Bonds in reaction systems were identified using a custom Python program that analyzes atom types and interatomic distances, determined through the results of radial distribution function (RDFs)^47^ with a cutoff distance of 0.45 Å. The visualization of simulation results was assisted with OVITO^60^ and the Python 2D graphics package Matplotlib.^61^

## Results and Discussion

### Surface-Adsorbed Species

In the LAO process, the action of an electric field typically causes water molecules to dissociate, leading to the adsorption of −OH and −H on silicon substrates. This is expected to alter the surface hydrophilicity and/or hydrophobicity,^62^ as well as lead to different degrees of oxide growth.^63^ This work compares the simulation results on three silicon surfaces (100), (110), and (111) before and after bias-induced oxidation with applied electric fields at a strength of 6 V/nm. This particular strength was selected based on a previous study,^47^ which demonstrated that it could effectively enhance oxidation without excessively consuming water during the 1 ns oxidation period.

**Figure 2:**
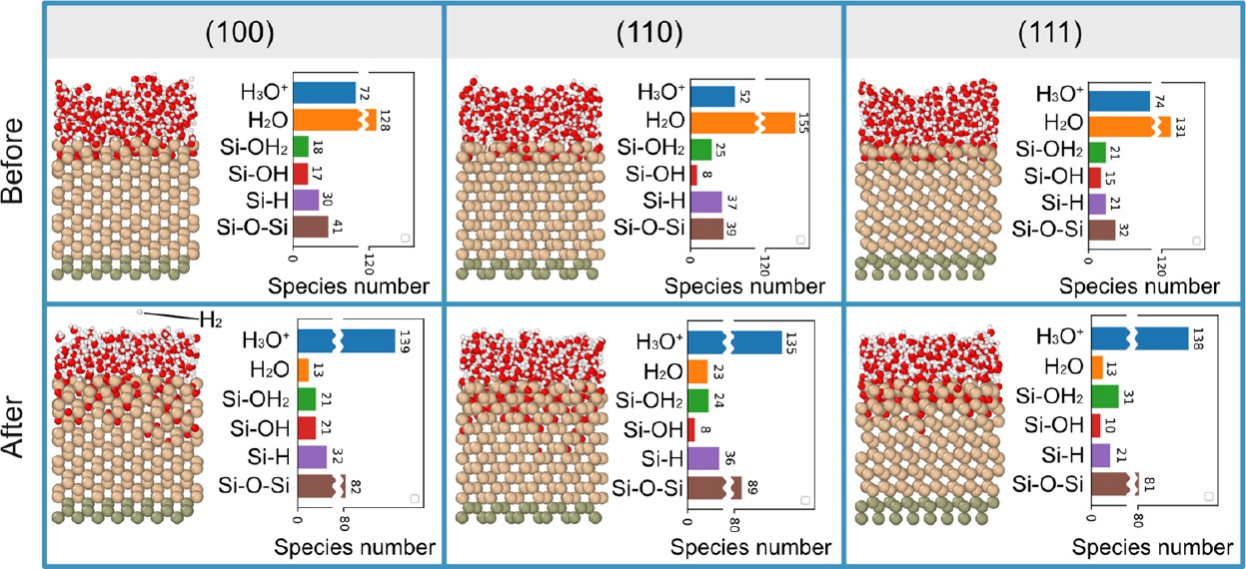
Snapshots of water-silicon systems in the simulation and species counting before and after bias-induced oxidation on silicon (100), (110), and (111) surfaces.

Figure 2 illustrates the results of surface passivation and bias-induced oxidation on three different substrate surfaces. The involved bonds and species were counted using a Python program based on the interatom distances. Compared with a pristine silicon surface, a water-silicon reaction under and without an electric field leads to the consumption of water and the creation of numerous bonds or species. In particular, H_2_O was found to adhere to the silicon surface through molecular adsorption to form Si–OH_2_ or through dissociative adsorption to form Si–OH, agreeing with previous studies.^37,64^ As a result, all three types of silicon surfaces are covered with −OH, −H, and −OH_2_. The oxide layer is dominated by Si–O–Si and Si–Si (suggesting a SiO_*x*_ composition^65^), and the resultant water films consist of H_3_O^+^ (hydronium) and H_2_O. Moreover, the comparison of results before and after bias-induced oxidation in Figure 2 shows that the application of electric fields apparently enhances the oxidation by creating more Si–O–Si bonds and consuming more water.

**Figure 3:**
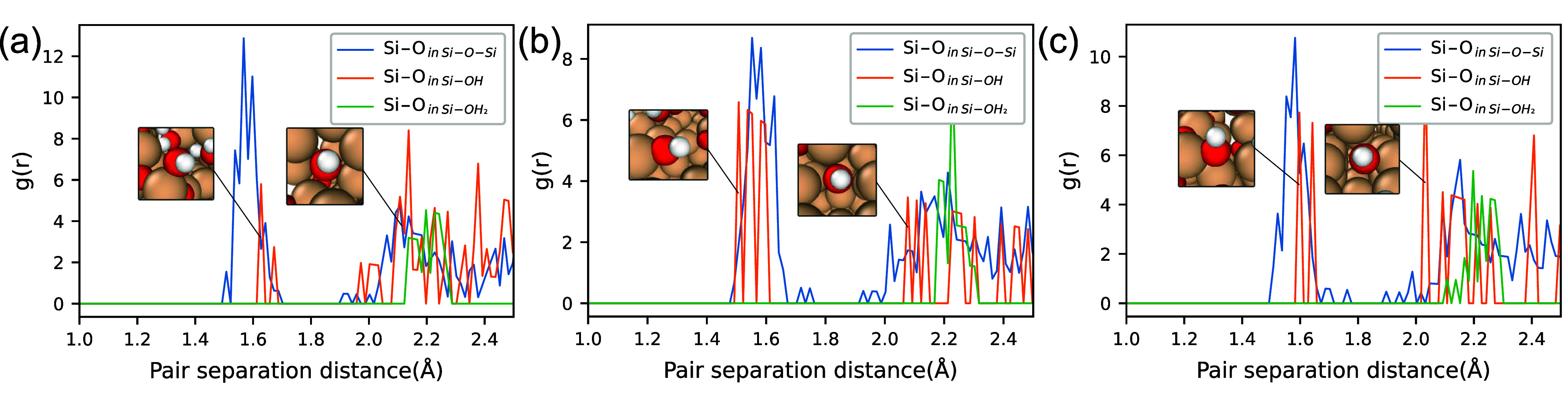
Calculated RDFs of Si–O pair in Si–O–Si, Si–OH, and Si–OH_2_ on (a) (100), (b) (110), and (c) (111) surfaces after bias-induced oxidation.

**Figure 4:**
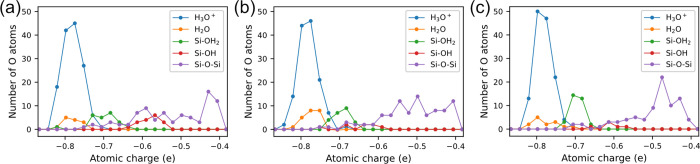
Distribution of O atoms at different atomic charges for bias-induced oxidation results on silicon (a) (100), (b) (110), and (c) (111) surfaces.

To reveal the structure of these adsorbed species, Figure 3 plots the RDFs of the Si–O pairs in Si–OH, Si–O–Si, and Si–OH_2_ for bias-induced oxidation results in three surfaces. For RDFs on all three surfaces, a majority of Si–O in Si–O–Si bonds appear near the distance of 1.6 Å, agreeing with the Si–O bond length in silica.^66^ The pair separation distance of Si–O in H_2_O–Si is located between 2.18 and 2.31 Å. This indicates that surface-adsorbed H_2_O is positioned further from the surface Si atoms compared to the Si–O bond length, aligning with the structure of the Si–OH_2_ pair (2.3 Å) in a previous report.^67^Figure 4 plots the charge of O atoms in different species or bonds. O in H_2_O carried a greater negative charge than that in Si–OH_2_. This indicates that the formation of Si–OH_2_ involves partial donation of charge from O atoms to Si atoms, agreeing with the conclusion in a previous study.^67^ Si–O in Si–OH is located differently, as shown in the insets in Figure 3, in which −OH can be located at the interatom position between Si atoms or attached to one Si atom. These two locations lead to different Si–O pair distances near 1.6 or 2 Å, close to previous first-principles studies.^68−70^ The formation of Si–OH involves a further charge transfer because the O atoms in them occupy lower charges than Si–OH_2_, as shown in Figure 4.

**Figure 5:**
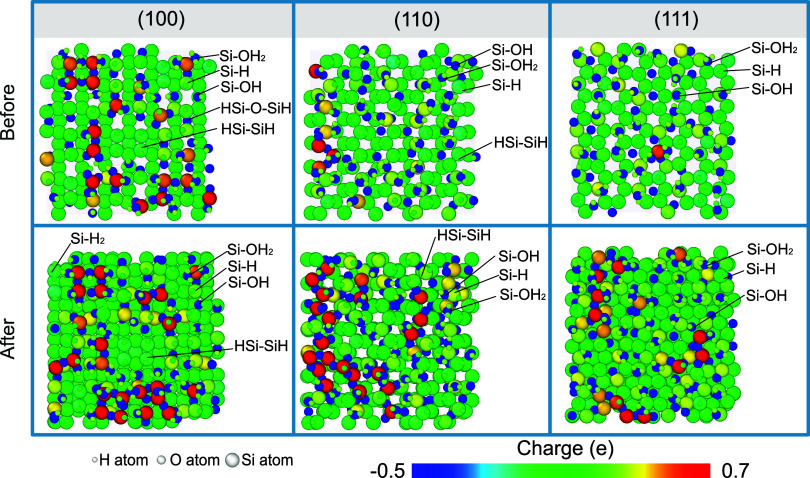
Snapshots of oxidized silicon surfaces (after removing H_2_O/H_3_O^+^) for bias-induced oxidation with an electric field strength of 6 V/nm on silicon (100), (110), and (111) surfaces. Atoms are colored by Mulliken charges, with different sizes representing different atom types.

Surface-adsorbed groups and charge distribution on different surfaces are not exactly the same. Figure 5 illustrates the surfaces before and after bias-induced oxidation after removing water molecules and hydronium ions that maintained their molecular forms so that only silicon atoms and surface terminations as −OH_2_, −OH, and −H remain in view. It should be noted that the calculated charges are determined from the electronegativity equalization method (EEM), fitted to Mulliken charges.^71^ For all three surfaces, −OH_2_, −OH, and −H were found on different surfaces before and after bias-induced oxidation. These surface adsorptions may assemble to form HSi–SiH and HSi–O–SiH structures, particularly on the (100) and (110) surfaces, as noted in Figure 5. Only the (100) surface was found with dihydrated Si atoms. These results are in accordance with previous experimental and simulation studies for silicon wet oxidation and chemical mechanical polishing.^37,72−75^ Due to the hydrophobic properties of Si–H bonds caused by small polarity, −OH_2_ and −OH were not observed near Si–H sites but mostly appeared at or near the sites of bare surface Si atoms or Si atoms bonded with O atoms underneath. Figure 5 also shows that the bias-induced oxidation on the Si(100) surface can generate H_2_ as byproducts. Although only 1 to 2 H_2_ molecules were detected throughout our entire simulation, this is still a typical occurrence during surface oxidation.^76^

**Figure 6:**
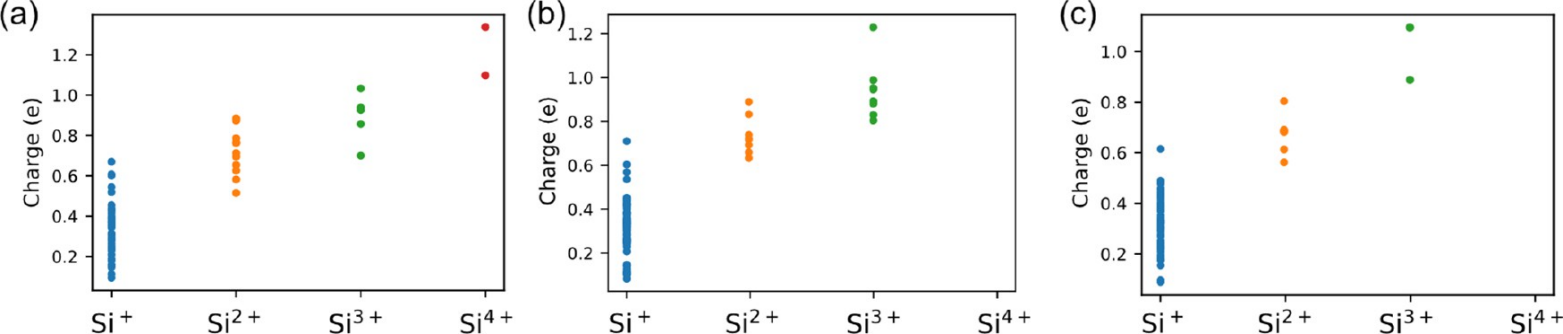
Atomic charge plot of Si^+^, Si^2+^, Si^3+^, and Si^4+^. Results were calculated from simulation results for (a) (100), (b) (110), and (c) (111) surfaces under the electric field with 6 V/nm strength.

As a result of surface passivation and bias-induced oxidation, silicon surfaces in Figure 5 were apparently oxidized, as evidenced by a significant number of Si atoms carrying positive charges. Moreover, noticeably more Si atoms were oxidized during the bias-induced oxidation process. Figure 5 shows that Si atoms on the surface could be oxidized or reduced when they are attached to different species. In particular, the Si atom in Si–H could show a negative charge due to the strong polarization toward the Si atom. For Si–OH_2_, Si–OH, and Si–O–Si, O atoms in these bonds/species, occupying different levels of negative charge as shown in Figure 4, typically make the Si atoms with positive charges, i.e., oxidized. Figure 6 displays the atomic charges of different types of Si atoms, categorized by the number of neighboring O atoms for the Si–O bond length of 1.58 Å, showing results from bias-induced oxidation on three different surfaces. Here, we define Si atoms with 1, 2, 3, or 4 neighboring O atoms as Si^+^, Si^2+^, Si^3+^, and Si^4+^. We can see that the charge of Si atoms increases with the number of neighboring O atoms, which is also illustrated in the charge plot in Figure 5. This occurs because O atoms have a stronger negative charge relative to Si atoms, causing Si atoms to become more positively charged as the number of neighboring O atoms increases. For the Si atom neighboring four O atoms, its charge can reach close to 1.2 *e*. This charge value is consistent with the Si atom under similar neighboring conditions, as demonstrated by ab initio calculation results and ReaxFF MD simulation outcomes.^77,78^

Figure 6 also indicates a higher concentration of Si^3+^/Si^4+^ atoms on the (100) surface compared to that on the (110) surface, and the least on the (111) surface; notably, Si^4+^ atoms are absent on (110) and (111) surfaces. This distribution is likely influenced by atomic density, as the (100) surface has the lowest atomic density among the three, while the (111) surface features a more densely packed structure than the (110) surface in its first two layers. This suggests that the oxidation and elevation of Si atoms on the (111) surface is more challenging due to its structural configuration.

### Bias-Induced Oxidation Processes

**Figure 7:**
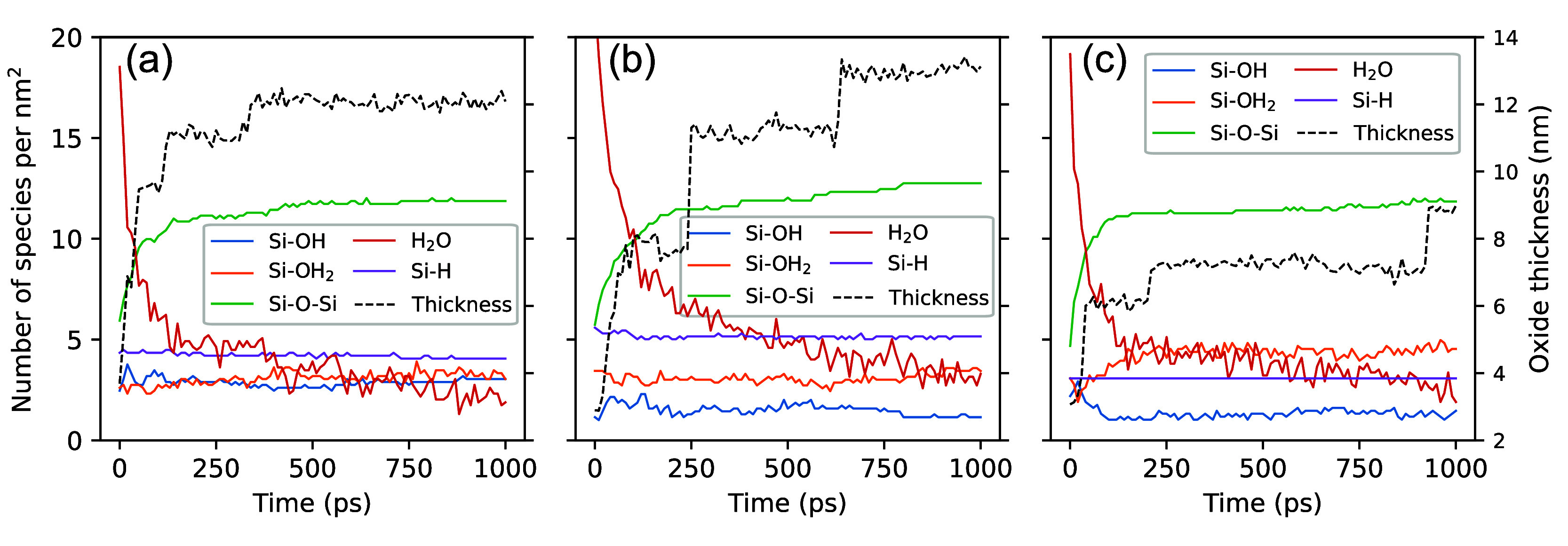
Evolution of species per unit area (1 nm^2^) and oxide thicknesses during bias-induced oxidation on (a) (100), (b) (110), and (c) (111) surfaces.

The bias-induced oxidation processes on three crystal orientations can be examined by analyzing the bond/particle number changes in Figure 7. In accordance with a previous study,^51^ bias-induced oxidation on all three surfaces mainly involves the consumption of water and the creation of Si–O–Si bonds and H_3_O^+^. The numbers of −OH_2_, −OH, and −H on silicon (100) and (110) surfaces remain nearly the same before and after bias-induced oxidation.^47,48^ However, the (111) surface underwent a noticeable increase in the number of −OH_2_ and a decreased number of −OH. Interestingly, the total number of them basically remains the same. The comparison of the positions of −OH_2_ and −OH in Figure 5 shows that they occupy the same sites on the (111) surface. This indicates that the action of the electric field consumed −OH and created the vacancies filled by −OH_2_. These results demonstrate that bias-induced oxidation occurs differently depending on surface orientations, leading to different surface compositions. As a result, both silicon (100) and (110) are dominated by H-terminations, while the (111) surface is dominated by adsorbed H_2_O, which could be the reason for the high porous oxide formed during the LAO process.^79^ The silicon (100) surface contains more Si–OH than the other two surfaces, which agrees with experimental observations for silicon surfaces in anodic solutions.^80^ The numbers of Si–H on three surfaces are not apparently affected during bias-induced oxidation, particularly on the (111) surface. The higher binding energy of Si–H compared to Si–Si could explain why modifications to Si–Si bonds play a dominant role during bias-induced oxidation.^81,82^ Due to the Si atom density on the (100) surface being the lowest among the three, −OH has more adsorption sites within a wider range of depth on the surface, and that could be the reason why the (100) surface contains more Si–OH than the other surfaces.

**Figure 8:**
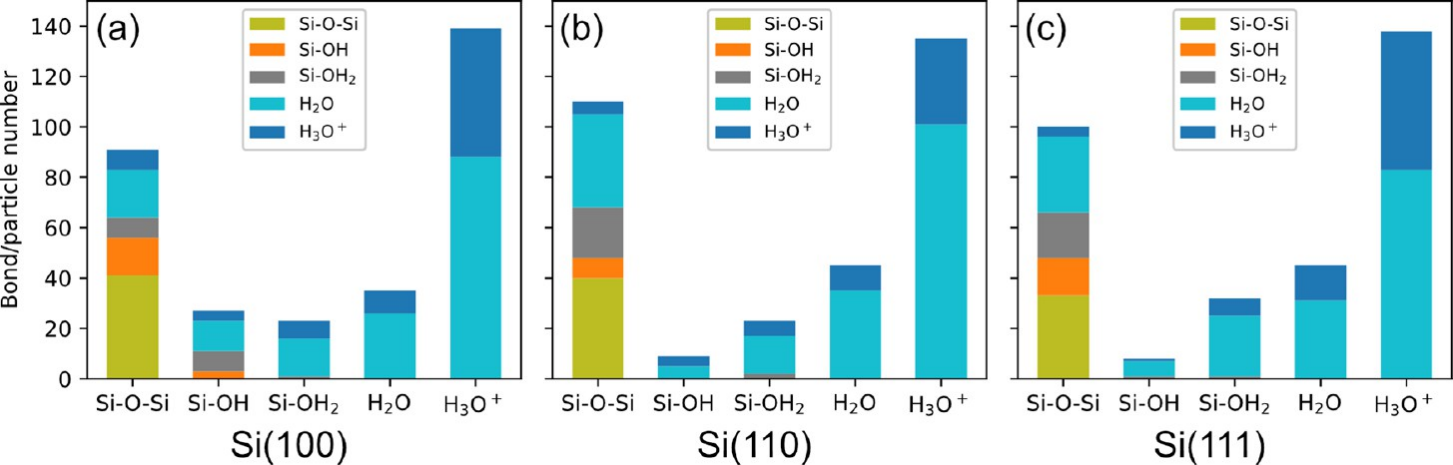
Bar chart showing the bond/particle number of Si–O–Si, Si–OH, Si–OH_2_, H_2_O, and H_3_O^+^ of bias-induced simulation results on silicon (a) (100), (b) (110), and (c) (111) surfaces. The bars are color-coded to represent O atoms in each bond/species type in surface passivation results.

**Figure 9:**
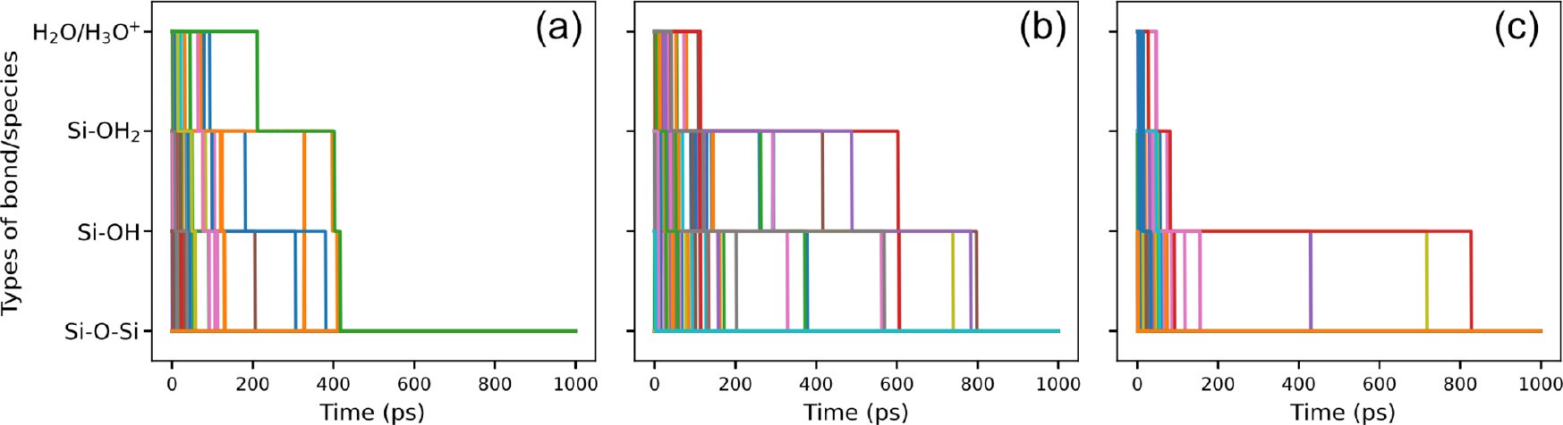
Evolution of bond types/species associated with O atoms in Si–O–Si bonds during surface bias-induced oxidation on silicon (a) (100), (b) (110), and (c) (111) surfaces.

**Figure 10:**
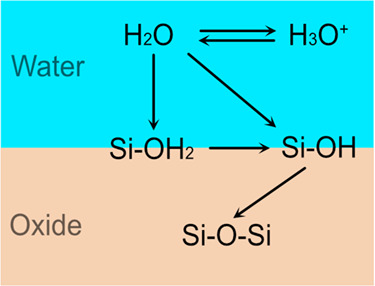
Schematic of O atom flow during bias-induced oxidation on silicon (a) (100), (b) (110), and (c) (111) surfaces.

To uncover more details of the reaction process, we tracked all the O atoms before and after bias-induced oxidation by identifying their belongings to Si–O–Si, −OH, −OH_2_, or H_2_O/H_3_O^+^ using a self-developed Python program. The findings, plotted in Figure 8, show that O atoms in Si–O–Si in bias-induced oxidation results can originate from any of the bonds/species containing O atoms prior to bias-induced oxidation. O atoms in −OH may originate from −OH, −OH_2_, and H_2_O/H_3_O^+^ while those in −OH_2_ may only come from −OH_2_ and H_2_O/H_3_O^+^. These observations indicate a directional flow of O atoms throughout the bias-induced oxidation. To further verify this, we also tracked the evolution of bond/species types to which the O atoms in Si–O–Si bonds belong during surface bias-induced oxidation. This tracking was conducted using a time step of 0.1 ps to ensure accuracy and to prevent any oversight of O atom migration to other species during this period. These results are plotted in Figure 9 which demonstrated that O atoms typically flow through different species or bonds before forming the Si–O–Si bonds on three silicon surfaces. During bias-induced oxidation, most O atoms in Si–O–Si bonds (in results) originate from H_2_O/H_3_O^+^, progress to −OH_2_, then to −OH, and finally to Si–O–Si, as plotted in Figure 10. Combined with previous studies,^42,47^ following reaction steps during the bias-induced oxidation were demonstrated. First, H_2_O adsorbs to the silicon surface, forming a Si–OH_2_ (molecular adsorption):
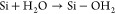
1

Next, the Si–OH_2_ bond can dissolve into Si–OH bond, and the released H atom combined with H_2_O to form H_3_O^+^:
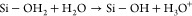
2

It is possible that water can directly react with surface silicon to form Si–OH, which represents the dissociated adsorption:^64^
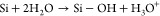
3

Si–OH can react with one of the surrounding Si atoms to form a Si–O–Si bond, and the released H atom combined with H_2_O again to form H_3_O^+^:
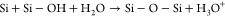
4

Therefore, H_2_O molecules can be consumed step by step during these reactions, eventually forming the Si–O–Si bonds. In our simulation, we found that −OH_2_ and −OH are positioned at the water-oxide interface, acting as intermediate products.^47^ These concluded reactions comply with previous theoretical studies, first-principles calculations, and scanning tunneling microscopy observations.^74,83−87^ In addition, no atom exchange was observed in the opposite way in our simulation results, e.g., atom flowing from Si–O–Si to −OH, then to −OH_2_, indicating these concluded reactions are not reversible. As for O atoms in H_3_O^+^ and H_2_O, O atom exchange can easily happen due to the proton transfer, which has already been confirmed by the ab initio MD simulation study.^88^

From these reactions, we know that the numbers of −OH_2_ and −OH can determine the rate of the bias-induced oxidation process. However, it remains unclear which specifically dictates the oxidation rate. Analyzing the evolution of −OH groups on three surfaces in Figure 7, particularly within the first 100 ps of bias-induced oxidation, we observe high generation rates of Si–O–Si bonds and rapid increases in oxide thickness, indicating accelerated oxidation rates on these surfaces. During this period, the increase in oxidation rate correlates with an increased number of Si–OH group, suggesting that this functional group play a crucial role in enhancing oxidation,^89^ especially pronounced on the (110) and (111) surfaces. When the number of Si–OH drops, the oxidation apparently slows. This provides evidence that the bias-induced oxidation rate depends on the number of surface-adsorbed Si–OH species to form Si–O–Si bonds (reaction 4). This conclusion agrees with previous theoretical investigations, indicating that the oxidation reaction is dominated by the production of −OH for the moderate oxide thickness and exposure time.^63,86^ In addition, bias-induced oxidation on the (100) surface appears to be less influenced by the quantity of Si–OH groups. This may be attributed to its significantly higher count—nearly double—that of (110) and (111) surfaces, which likely provides an ample supply for sustained oxidation. Figure 7 does not indicate an observable correlation between the oxidation rate and the quantity of −OH_2_ groups. This could be due to their numbers exceeding those of −OH, facilitating the rapid occurrence of reaction 2.

### Oxide Growth

Previous simulation studies^42,43,47^ have typically assessed oxide growth by measuring the *z*-distance between the top Si atom and the bottom O atom, providing a straightforward oxide thickness evaluation method. However, this approach can overlook scenarios when negatively charged O ions migrate below the surface without significantly enhancing surface oxidation, marked by no notable increase in Si–O–Si bonds and no additional water consumption. This oversight can lead to unwanted results when evaluating the oxidation process under strong electric fields.^47^ In this ReaxFF MD simulation study, we aim for a thorough analysis of oxidation. The oxide growth is evaluated by tracking changes in the number of Si–O–Si bonds per nm^2^ and monitoring the surface elevation as oxidation proceeds. In addition, straightforward oxide thickness is also monitored to facilitate comparisons.

**Figure 11:**
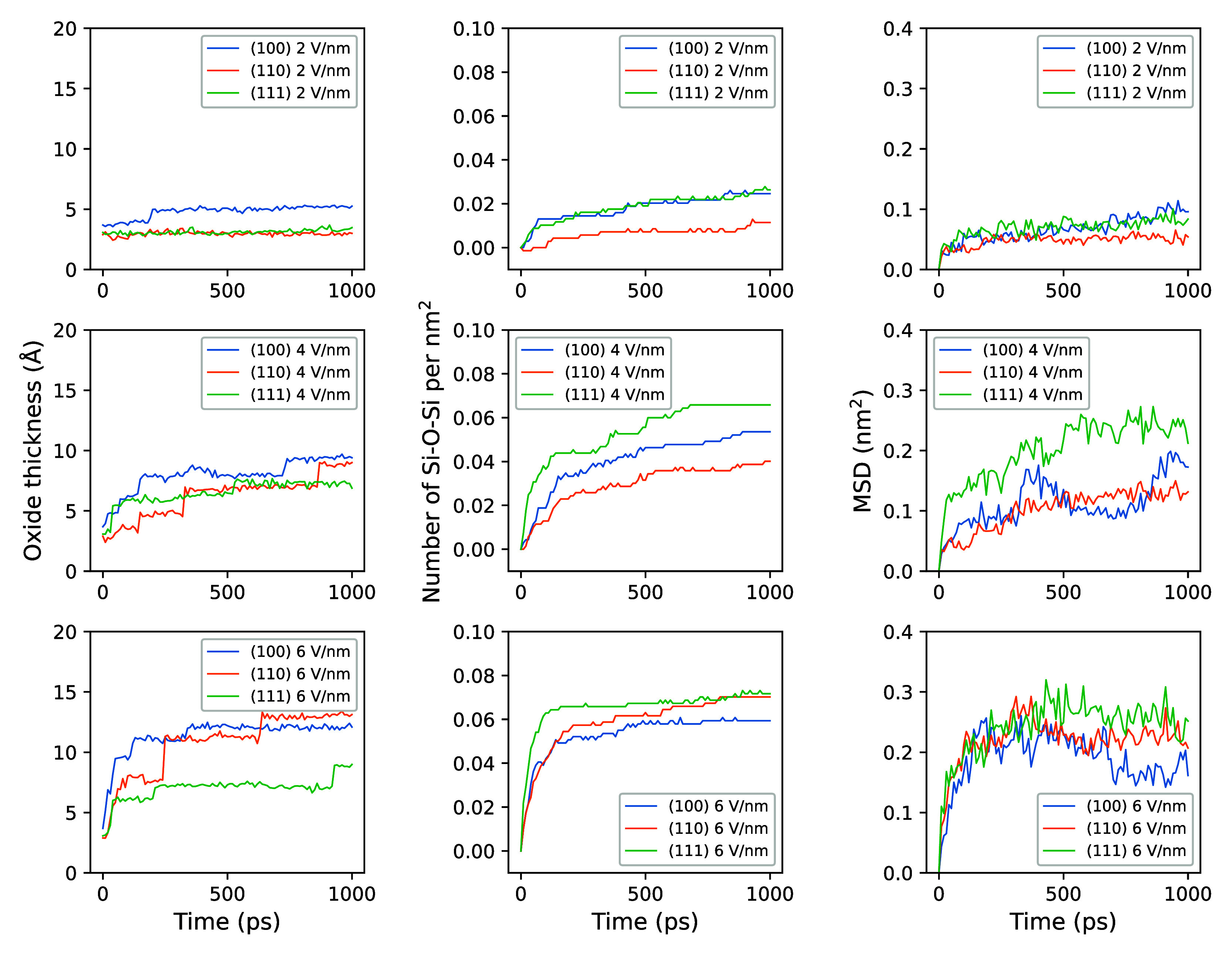
Comparisons of the evolution of oxide thickness, number of Si–O–Si per nm^2^, and MSDs during the bias-induced oxidation using electric field strengths of 2, 4, and 6 V/nm.

To examine the effect of electric field strength, we applied electric fields with strengths of 2, 4, and 6 V/nm, respectively, in simulation and presented the comparative results in Figure 11. The surface elevation induced by bias-induced oxidation is reflected by the calculation of mean square displacement (MSD) of surface Si atoms before and after bias-induced oxidation, following the equation:
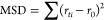
5For atom *i*, the initial position is *r*_0,_ and the time *t* position is *r*_*ti*_.

Figure 11 first shows the evolution of oxide thicknesses during each group of simulations. Apparently, the (100) surface typically has a thicker oxide layer, except when subjected to oxidation using a 6 V/nm electric field, under which the (110) surface develops a thicker oxide. This phenomenon could stem from the lower atomic density of the (100) surface, which aids the diffusion of negatively charged O atoms within Si–O–Si bonds, thereby introducing more O atoms deep under the silicon surface in Figure 5. However, oxide thickness alone may not accurately represent the oxide growth rate, as it has no obvious correlation with the creation of Si–O–Si bonds or consumption of H_2_O. Regarding the generation rate of Si–O–Si bonds, Figure 11 indicates that the (111) surface exhibits a markedly higher rate compared to the (100) surface. As the electric field strength increases to 6 V/nm, the Si–O–Si bond formation rate on the (110) surface accelerates, surpassing that on the (111) surface at 1 ns. Similarly, the evolution of MSDs also demonstrated that the MSD on the (110) surface increases with elevated electric field strengths, approaching that of the (111) surface and surpassing the (100) surface at 1 ns. These analyses demonstrate a good correlation between increased Si–O–Si bond formation and elevated surface growth. This could suggest a more reliable method for evaluating oxide growth compared to simply measuring oxide thickness.

Given that our simulations are limited to the initial stages of oxidation, encompassing only a few atomic layers in thickness, the role of Si atomic density becomes critical. Higher atomic densities on the surface facilitate the adsorption of more O atoms, thereby enhancing the oxidation process.^90^ However, a higher atomic density, at the same time, limits the ability of O atoms to pass through the atomic layer. In particular, the (111) surface has a special bilayer stacked structure, with the first two layers distancing at only 0.077 nm, much smaller than the distance between the second and the third 0.23 Å, as shown in Table 1. As a result, it is more difficult for O atoms to diffuse through the closely packed bilayers than the regularly spaced Si layers on (110) and (100) surfaces. This gives a reasonable explanation for a slow diffusion of O atoms on the (111) surface.

Integrating the discussed effects, we observe distinct anisotropic behaviors during the initial stages of in the ReaxFF simulation of bias-induced oxidation. The silicon (111) surface demonstrates a superior initial oxide growth trend (within the first 100 ps) compared to the other orientations, likely attributed to its close-packed bilayer structures that result in the highest atomic density within the oxide. The silicon (110) surface exhibits a greater oxide growth rate under high-strength electric fields due to its high single-layer atomic density and proper atom permeability. Conversely, the silicon (100) surface experiences the slowest oxide growth rate, primarily due to its lower atomic density.

## Conclusions

In this work, ReaxFF MD simulations were performed to investigate bias-induced oxidation on silicon (100), (110), and (111) surfaces, aiming to elucidate the anisotropic effects inherent in the LAO process. The application of an electric field resulted in varied oxidation behaviors across these surface orientations. Specifically, the (111) surface experienced a notable increase in −OH_2_ and a decrease in −OH, in contrast with the unchanged conditions observed on the (100) and (110) surfaces. The action of an electric field accelerates surface oxidation by increasing the number of oxygen atoms below the surface and enhancing the positive charge on silicon atoms. This effect is particularly pronounced on the (100) surface due to its lower atomic density. Furthermore, −OH_2_ and −OH were found to serve as intermediate products during the silicon bias-induced oxidation, with the quantity of −OH directly influencing the oxidation rate. Although determining the exact LAO reaction rate remains challenging, initial bias-induced oxidation stages suggest a higher reaction rate on the (111) surface among the three surfaces, with the (110) surface reacting more swiftly under higher electric fields. These findings and conclusions agree well with previous theoretical and experimental studies.

Our simulation outcomes could shed light on the chemical composition and charge states of LAO-enabled silicon nanostructures, as well as their atomic-level dynamics, offering insights for more precise process control to achieve desired silicon surface structures. Moreover, integrating in situ detection of Si–OH species may provide a novel monitoring method for the LAO process. These insights offer a promising approach to advancing LAO nanolithography toward a reliable nanofabrication method for silicon nanodevices.

## Data Availability

All data underpinning this publication are openly available from the University of Strathclyde Knowledge Base at: 10.15129/ccd5c042-7c58-4b40-953f-8725b6d71624.
